# MSPminer: abundance-based reconstitution of microbial pan-genomes from shotgun metagenomic data

**DOI:** 10.1093/bioinformatics/bty830

**Published:** 2018-09-25

**Authors:** Florian Plaza Oñate, Emmanuelle Le Chatelier, Mathieu Almeida, Alessandra C L Cervino, Franck Gauthier, Frédéric Magoulès, S Dusko Ehrlich, Matthieu Pichaud

**Affiliations:** 1 Enterome, 94-96 Avenue Ledru Rollin, Paris, France; 2 MGP MetaGénoPolis, INRA, Université Paris-Saclay, Jouy en Josas, France; 3 CentraleSupélec, Université Paris Saclay, 9 rue Joliot Curie, Gif-sur-Yvette, France

## Abstract

**Motivation:**

Analysis toolkits for shotgun metagenomic data achieve strain-level characterization of complex microbial communities by capturing intra-species gene content variation. Yet, these tools are hampered by the extent of reference genomes that are far from covering all microbial variability, as many species are still not sequenced or have only few strains available. Binning co-abundant genes obtained from *de novo* assembly is a powerful reference-free technique to discover and reconstitute gene repertoire of microbial species. While current methods accurately identify species core parts, they miss many accessory genes or split them into small gene groups that remain unassociated to core clusters.

**Results:**

We introduce MSPminer, a computationally efficient software tool that reconstitutes Metagenomic Species Pan-genomes (MSPs) by binning co-abundant genes across metagenomic samples. MSPminer relies on a new robust measure of proportionality coupled with an empirical classifier to group and distinguish not only species core genes but accessory genes also. Applied to a large scale metagenomic dataset, MSPminer successfully delineates in a few hours the gene repertoires of 1661 microbial species with similar specificity and higher sensitivity than existing tools. The taxonomic annotation of MSPs reveals microorganisms hitherto unknown and brings coherence in the nomenclature of the species of the human gut microbiota. The provided MSPs can be readily used for taxonomic profiling and biomarkers discovery in human gut metagenomic samples. In addition, MSPminer can be applied on gene count tables from other ecosystems to perform similar analyses.

**Availability and implementation:**

The binary is freely available for non-commercial users at www.enterome.com/downloads.

**Supplementary information:**

Supplementary data are available at *Bioinformatics* online.

## 1 Introduction

Metagenomics has revolutionized microbiology by allowing culture-independent characterization of microbial communities. Its advent has allowed an unprecedented genetic characterization of the human gut microbiota and emphasized its fundamental role in health and disease (Wang *et al.*, 2015). Shotgun metagenomics where whole-community DNA is randomly sequenced bypasses the biases and limitations of 16S rRNA sequencing (Brooks *et al.*, 2015; Větrovský and Baldrian, 2013) by providing high resolution taxonomic profiling as well as insights into the diverse physiological roles and the metabolic potential of the community (Jovel *et al.*, 2016; Ranjan *et al.*, 2016).

The analysis of large cohorts revealed a substantial inter-individual microbial gene content variability (Li *et al.*, 2014), nucleotide polymorphism (Schloissnig *et al.*, 2013) which reflects that individuals are not only carriers of various species, but also of different strains of the same species (Greenblum *et al.*, 2015; Zhu *et al.*, 2015). The characterization of the accessory genes found in individual strains is crucial in many contexts as they can provide functional advantages such as complex carbohydrates metabolism (Larsbrink *et al.*, 2014), antibiotic resistance or pathogenicity (Loman *et al.*, 2013; Scaria *et al.*, 2010).

Recent analysis toolkits for shotgun metagenomics data achieved strain-level resolution when coverage is sufficient. To this end, they either capture intra-species single-nucleotide polymorphisms (SNPs) in pre-identified marker genes (Luo *et al.*, 2015; Truong *et al.*, 2017), gene content variation (Scholz *et al.*, 2016) or both (Nayfach *et al.*, 2016). However, these tools are hampered by the extent of sequenced genomes.

Indeed, microbial variability extends far beyond the content of available genomes making metagenomic samples an untapped reservoir of information. First, it has been estimated that on average 50% of the species present in the human gut microbiota of Western individuals lack reference genome and this proportion rises to 85% in individuals with traditional lifestyles (Nayfach *et al.*, 2016). Even if recent advancements of culture-based methods have proven that a substantial proportion of these species are actually cultivable (Browne *et al.*, 2016; Lagier *et al.*, 2016), the number of unknown species is probably still important. In addition, these techniques remain laborious and time consuming. Second, although species of public health interest (e.g. *Escherichia coli*, *Salmonella enterica* or *Clostridium difficile*) are represented by hundreds or even thousands of genomes in public databases, only few strains are available for the great majority of commensal species. Consequently, accessory genes associated with microbial phenotypic traits may be missing in gene repertoires constructed from reference genomes.

*De novo* metagenomic assembly where overlapping reads are merged into longer sequences called contigs is a powerful reference-free technique for overcoming the limitations of reference-based methods. However, assembly remains a computationally challenging task and despite the many dedicated tools proposed, the process only recovers incomplete genomes scattered in multiple contigs (Sczyrba *et al.*, 2017). In an attempt to obtain exhaustive references, metagenomic assembly is performed on multiple samples to create non-redundant gene catalogs (Almeida and Pop, 2015).

Subsequently, these catalogues are used in metagenome-wide association studies for disease-related analyses (Wang and Jia, 2016) or descriptive purposes (Li *et al.*, 2014). However, testing millions of genes is biased towards organisms with the most genes in the pool as they have more chances of being picked up. In addition, this approach lacks statistical power because many genes have strongly correlated abundances profiles which amounts to perform the same test multiple times (Schwartzman and Lin, 2011).

Considering that the physically linked genes should have proportional abundances across samples, binning co-abundant genes has been proposed to organize catalogs into clusters of genes originating from the same biological entity. However, clustering millions of genes is a challenging task as pairwise comparison of all gene abundance profiles is computationally intensive. To reduce the number of comparisons, some authors have performed binning of the subset of genes that were statistically significant by themselves (Le Chatelier *et al.*, 2013; Qin *et al.*, 2012), which does not improve the statistical power of the analysis. Others have proposed methods to perform the clustering of complete gene references based either on the Markov clustering algorithm (Karlsson *et al.*, 2013), the Chameleon clustering algorithm (Jie *et al.*, 2017) or a variant of the Canopy clustering algorithm (Nielsen *et al.*, 2014).

Although direct proportionality is expected between co-abundant genes, these methods rely either on Pearson’s or Spearman’s correlation coefficients which respectively assess a linear association with a potentially non-null intercept or any monotonic association. Thus, these coefficients are not specific enough and spurious associations can be discovered. In addition, they are hampered by rare genes with many null counts (Huson, 2007), non-normal gene counts distributions (Kowalski, 1972) and presence of outliers (Osborne and Overbay, 2004).

Furthermore, current clustering strategies group species core genes and highly prevalent accessory genes into the same cluster, but miss lower prevalence accessory genes or assign them to small separate clusters (Almeida *et al.*, 2016). Dependency between core and accessory clusters can be evaluated downstream using the Fisher’s exact test (Nielsen *et al.*, 2014), which compares their presence/absence patterns across samples. Yet, this strategy does not account for the co-abundance of genes and is poorly discriminative when considering accessory clusters that are rare or associated with very prevalent species. In addition, it is not suitable for detecting genes shared between several species.

To overcome these limitations, we developed MSPminer, the first tool that discovers, delineates and structures Metagenomic Species Pan-genomes (MSPs) from large-scale shotgun metagenomics datasets without referring to genomes from isolated strains. MSPminer presents several significant improvements over existing methods. First, it relies on a robust measure of proportionality for the detection of co-abundant but not necessarily co-occurring genes as expected for non-core genes. Second, genes grouped in a MSP are empirically classified as core, accessory or shared.

We applied MSPminer to the largest publicly available gene abundance table which is composed of 9.9 M genes quantified in 1267 human stool samples (Li *et al.*, 2014). We show that MSPminer successfully groups known and additional genes from species and that this information can be used for qualitative and quantitative analyses.

## 2 Materials and methods

### 2.1 Rationale behind MSPminer

Microbial pan-genomes are gene repertoires composed of core genes present in all strains and accessory genes present in only some of them (Medini *et al.*, 2005). In a shotgun metagenomic sequencing context, we define as shared the genes detected in some samples where the species is not present.

A strain found in a sample is an instance of the species pan-genome: it is made of all the species (shared) core genes and of a subset of (shared) accessory genes. Core genes are suitable for taxonomic profiling at species-level while accessory genes can be used to compare strains across samples. Genes tagged as shared should be used carefully as they contain false positives counts or are subject to horizontal transfer.

**Fig. 1.**
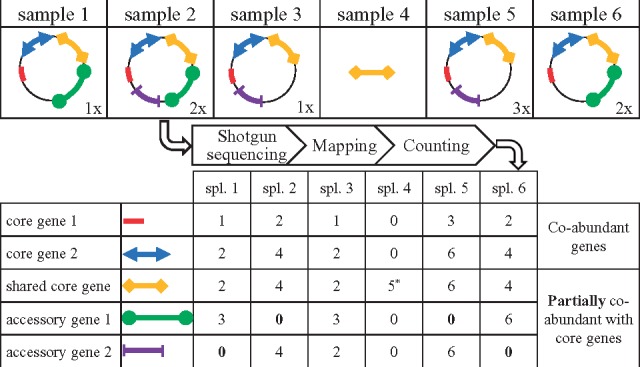
Simplified model illustrating the rationale behind the method. Six samples except the fourth carry a strain of a microbial species represented by a circle. The absolute abundance of each strain is indicated on the bottom right. Core genes (red, blue, yellow) are present in all the strains while accessory genes (green, purple) are found only in some. In addition, the yellow gene is tagged as shared because it is observed in sample 4 that do not contain the species. After shotgun sequencing, core genes yield directly proportional mapped reads counts across samples, the proportionality coefficient being roughly equal to the ratio of their length. In contrast, such relationship between a core and an accessory gene is observed only in the subset of samples where the accessory gene is present

We assumed that core genes of a microbial species should be consistently detected in samples where the species is present if sequencing depth allows (co-occurrence) and should yield directly proportional mapped reads counts across samples (co-abundance). Remarkably, a core and an accessory gene should have proportional counts only in the subset of samples carrying a strain with that accessory gene (Fig. 1).

### 2.2 Comparison of gene count profiles

To group the core genes of a species and then identify its accessory genes, we developed measures that detect pairs of genes with directly proportional counts even if this relationship occurs in a subset of samples.

Let $S=\left\{s_{1},s_{2},\ldots ,s_{m}\right\}$} be a set of m metagenomic samples. Let $g_{1}=\left(g_{1,s_{1}},g_{1,s_{2}},\ldots ,g_{1,s_{m}}\right)\;$and $g_{2}=\left(g_{2,s_{1}},g_{2,s_{2}},\ldots ,g_{2,s_{m}}\right)$ be the vectors of the number of mapped reads on the two genes to be compared. At first, the proposed method estimates a candidate coefficient of proportionality (α) between $g_{1}$ and $g_{2}$. Then, proportionality between $g_{1}$ and $g_{2}$ is assessed according to the coefficient α previously estimated ($p_{nr}$). Alternatively, proportionality is evaluated after outlier samples have been discarded ($p_{r}$).

In this study, count data is neither normalized by gene length, nor by read length nor by sequencing depth. Indeed, the number of times a gene is detected, which is the result of a stochastic process, is not accessible after normalization while it is needed for classifying null counts. Nonetheless, raw counts that follow in a first approximation a Poisson distribution were square root transformed to stabilize variance and reduce skewness (Bland and Altman, 1996).

#### 2.2.1 Estimation of the coefficient of proportionality

Suppose there is a relationship of proportionality between $g_{1}$ and $g_{2}$ noted $g_{2}=\alpha \cdot g_{1},\;with\;\alpha$ the coefficient of proportionality. α should be roughly equal to the ratio of the length $g_{2}$ by the length of $g_{1}$. However, this ratio is not always a good estimator, for instance when a gene is duplicated or when its coverage is non-uniform (Supplementary Fig. S1). Therefore, we robustly estimated α by calculating the median of the gene counts ratios:

$$\alpha =median\left(\frac{g_{2,s}}{g_{1,s}}\right)\;s\in S|\left(g_{2,s}\geq t\;{\wedge \;g}_{1,s}\geq t\right)\;\text{with}\;t=6$$

**Fig. 2.**
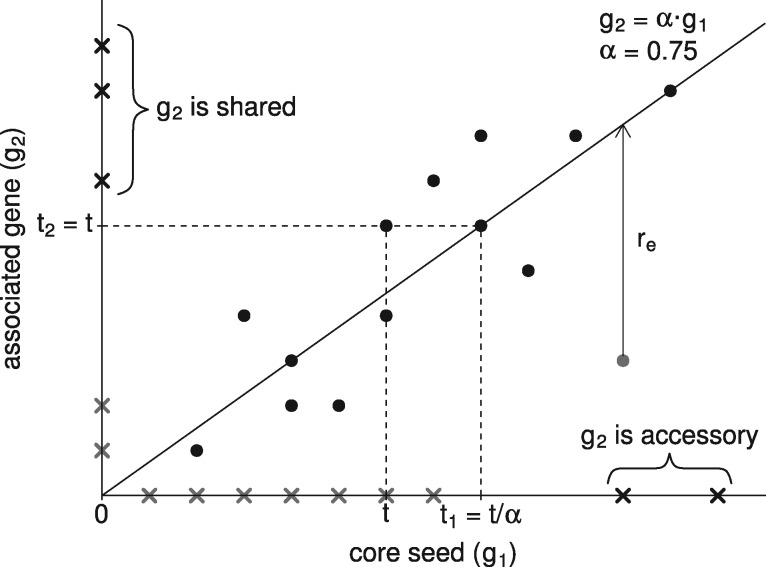
Method for comparing gene count profiles and classifying genes in MSPs. The counts of a gene ($g_{2}$) are compared to the counts of the core seed ($g_{1}$) with which it is associated across metagenomic samples. The coefficient of proportionality a between $g_{1}$ and $g_{2}$ is estimated to be 0.75. The solid line of slope α corresponds to expected counts. Dashed lines represent the gene quantification thresholds before and after adjustment according to α. Black and grey crosses are respectively structural and undetermined zeros. Only structural zeros are taken into account to assign $g_{2}$ to a given class (c.f. braces). Black and grey points are respectively inlier and outlier samples. The distance between the unique outlier and the expected proportional count correspond to the residual $r_{s}$

When estimating α, only samples where $g_{1}$ and $g_{2}$ had counts greater than a threshold t were taken into account (Fig. 2). This filtering has the following advantages:

1. It discards samples where both genes have null counts as they do not provide any quantitative information.
2. It discards samples where only one of the genes is present to allow detection of proportionality occurring in a subset of samples only.
3. It discards samples with low and scattered counts to allow a precise estimation of α.

#### 2.2.2 Classification of zeros

In a sample, a null count for a gene can be either a sampling or a structural zero. In the former situation, the gene is not detected because of sampling or technical artefacts, while in the latter the gene is really absent in the sample. Only accessory genes should yield structural zeros in samples where a microbial species is present. Thus, distinguishing these two kinds of zeros is crucial to accurately classify genes.

When a ≠ 1, different quantification thresholds for $g_{1}$ and $g_{2}$, respectively, named $t_{1}$ and $t_{2}$ where used to reflect that one gene has higher counts than the other:

$$t_{1}=max\left(t,\frac{t}{a}\right)\;and\;t_{2}=max\left(t,\alpha \cdot t\right)$$

Finally, a gene with a null count in a sample was classified as a structural zero if the other gene had a count greater than its threshold i.e. $\left(g_{2,s}\geq t_{2}{\;\wedge \;g}_{1,s}=0\right)$ or $\left(g_{2,s}=0{\;\wedge \;g}_{1,s}\geq t_{1}\right)$. Otherwise, it was classified as an undetermined zero (Fig. 2).

Assuming that count data follows a Poisson distribution, the probability of misclassifying a null count as a structural zero is 0.2% with an initial threshold t = 6 ($P\left(X\;=\;0||\lambda \;=\;6\right)=0.002$).

#### 2.2.3 Non-robust measure of proportionality – p_nr_

A modified version of the Lin’s concordance correlation coefficient (Lin, 1989) was used to estimate the agreement between $g_{1}$ and $g_{2}$ with a proportional relationship of coefficient α by using only samples where both genes had non-null counts:

$$p_{nr}=\frac{2\alpha \cdot cov(g_{1},g_{2})}{{\alpha \cdot \sigma }_{g_{1}}^{2}+\sigma_{g_{2}}^{2}+{\left(\alpha \cdot \bar{g_{1}}-\bar{g_{2}}\right)}^{2}}$$

where α is coefficient of proportionality previously estimated, $\bar{g_{1}}$ and $\bar{g_{2}}$ are the means, $\sigma_{g_{1}}^{2}$ and $\sigma_{g_{2}}^{2}$ are the variances and $cov(g_{1},g_{2})$ is the covariance of $g_{1}$ and $g_{2}$.

#### 2.2.4 Robust measure of proportionality – p_r_

We derived a robust version of the measure to identify associated genes despite the presence of samples with inconsistent counts, hereafter named outliers. This occurs for instance when multiple strains of a species coexist in the same sample.

First, the coefficient of proportionality α was estimated using the procedure previously described. Next, residuals ($r_{s}$) defined as the difference between observed and expected proportional counts were calculated on samples where both genes had counts above their respective quantification thresholds (Fig. 2):

$$R=\{r_{s}=g_{2,s}-\alpha \cdot g_{1,s}\}\;s\in S|\left(g_{2,s}\geq t_{2}\wedge g_{1,s}\geq t_{1}\right)$$

Then, the outliers (O) were detected using the Tukey’s method among the samples where both genes had non-null counts ($S^{'}$):

$$Q_{1}=1^{st}\_quartile\left(R\right)\;and\;Q_{3}=3^{rd}\_quartile(R)$$

$$IQR=\;Q_{3}-Q_{1}$$

$$lwr\_thr=Q_{1}-1.5\cdot IQR\;and\;upr\_thr=Q_{3}+1.5\cdot IQR$$

$$S^{'}=\{s\in S|\left(g_{2,s}>0\wedge g_{1,s}>0\right)\}$$

$$O=\{s\in S^{'}|\left(r_{s}<lwr\_thr\vee r_{s}>upr\_thr\right)\}\;and\;I=S^{'}\setminus O$$

Finally, the robust measure of proportionality $p_{r}$ was computed on inlier samples (I) using the same formula as $p_{nr}$. To avoid the detection of spurious associations with too many outliers, $p_{r}$ was not computed if $\left|O\right|>\left(\left|S^{'}\right|-5\right)\cdot 0.3$ that is to say a percentage of outliers greater than 30%.

### 2.3 Reconstitution of Metagenomic Species Pan-genomes

#### 2.3.1 Overview of MSPminer

We developed MSPminer, a clustering method that uses the measures of proportionality to group co-abundant genes into Metagenomic Species Pan-genomes (MSPs).

**Fig. 3.**
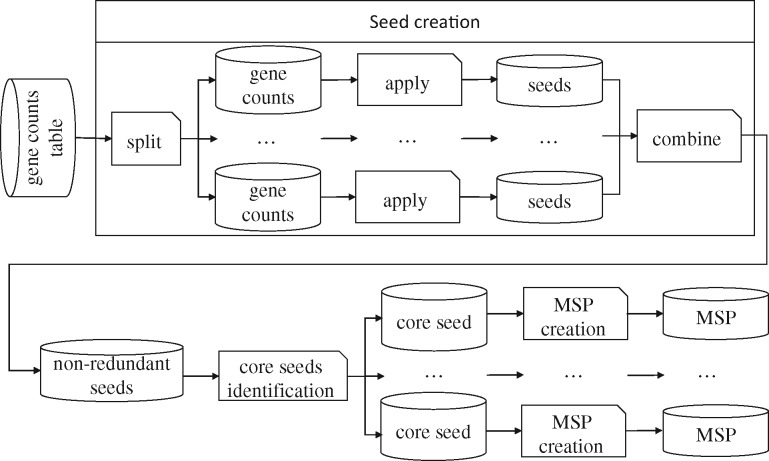
MSPminer workflow

MSPminer starts by identifying sets of directly proportional and co-occurring genes, called *seeds* using a split-apply-combine strategy. Then, seeds corresponding to species cores are empirically identified. Finally, all the genes associated to a core seed are grouped in a MSP where they are classified as (shared) core or (shared) accessory (Fig. 3).

#### 2.3.2 Input data and filtering

MSPminer takes as input a tab-separated values matrix giving the number of reads mapped on genes (rows) across metagenomic samples (columns). By default, only genes with counts greater than 6 in at least 3 samples were kept. Rarer genes were discarded because they do not support enough quantitative information for further processing.

#### 2.3.3 Seed creation

##### 2.3.3.1 Split

To avoid comparison of all pairs of genes, genes with the greatest count in the same sample were binned. This strategy not only decreases the number of comparisons to perform but increases the probability that related genes are placed in the same bin compared to random assignment (Supplementary Fig. S2). To achieve a good load balancing, raw read counts were normalized prior to bin assignment by the number of mapped reads in samples, as the procedure would be biased towards samples with high sequencing depth otherwise (Supplementary Fig. S3). This is the only step where normalized counts were used.

##### 2.3.3.2 Apply

Seeds were created in parallel in each bin with a greedy algorithm. First, all pairs of genes were compared and those with $p_{nr}$ greater than 0.8 and no structural zeros were saved in a list. Then, the list was sorted by decreasing $p_{nr}$ and the pair of genes with the greatest $p_{nr}$ was selected as a centroid. Genes related to one of the centroid genes ($p_{nr}$ ⩾ 0.8) were grouped together to form a seed and removed from the list. This procedure was iterated until the list was empty.

##### 2.3.3.3 Combine

Related genes might have been assigned to different bins, for instance when samples with the greatest counts had close values. Therefore, a merging step is required to generate a set of non-redundant seeds.

For each seed, a pseudo gene referred as *representative* was computed to compare seeds with each other. First, the seed representative was defined as the median vector of the counts of all the seed genes. Then, each gene of the seed was compared to the seed representative using $p_{nr}$. The final seed representative corresponded to the median vector of the counts of the 30 genes with the greatest $p_{nr}$ as these genes have the highest counts and the lowest dispersion.

Finally, seeds with $p_{nr}$ greater than 0.8 and no structural zeros were merged. After merging, seeds with less than 150 genes were discarded.

#### 2.3.4 Core seeds identification

Core seeds were identified among non-redundant seeds based on the assumption that in a set of related seeds, the largest corresponds to a species core and the others are modules of shared or accessory genes.

To this end, seeds were sorted by decreasing number of genes. The largest seed was defined as a new core seed. Then, the representative of the core seed was compared to the representative of all remaining seeds. Seeds with $p_{r}$ greater than 0.8 when compared to the core seed were discarded from the list of potential cores. The procedure was iterated until there was no more seed to process.

#### 2.3.5 Identification and classification of genes associated with a core seed

The representatives of each core seed were compared to all the genes. Genes with $p_{r}$ greater than 0.8 were considered as associated with the core seed. On real data, we found that this threshold is a good compromise between precision and sensitivity (Supplementary Fig. S4).

Let $g_{1}$ be the median vector of the number of mapped reads on a core seed and $g_{2}$ the vector of the number of mapped reads on a gene associated with this core seed. The associated gene was assigned to one of the four following classes according to the presence of structural zeros (Fig. 2):

1. Core: the gene was present in all the samples where core seed was detected and uniquely in those.
  $$\forall s\in S\;|\;\left(g_{1,s}\geq t_{1}\to \;g_{2,s}\neq 0\right)\wedge \left(g_{2,s}\geq t_{2}\to g_{1,s}\neq 0\right)$$
2. Accessory: the gene was present in a subset of samples where core seed was detected.
  $$\left(\exists s\in S\;|\;g_{1,s}\geq t_{1}\wedge g_{2,s}=0\right)\wedge \left(\forall s\in S\;{|\;g}_{2,s}\geq t_{2}\to g_{1,s}\neq 0\right)$$
3. Shared core: the gene was detected in all the samples where the core seed was present plus some samples where the core seed was absent.
  $$\left(\forall s\in S\;|\;g_{1,s}\geq t_{1}\to g_{2,s}\neq 0\right)\wedge \left(\exists s\in S\;|\;g_{2,s}\geq t_{2}\wedge g_{1,s}=0\right)$$
4. Shared accessory: the gene was detected in a subset of samples where the core seed was present plus some samples where the core seed was absent.
  $$\left(\exists s\in S\;|\;g_{1,s}\geq t_{1}\wedge g_{2,s}=0\right)\wedge \left(\exists s\in S\;{|\;g}_{2,s}\geq t_{2}\wedge g_{1,s}=0\right)$$

#### 2.3.6 Creation of Metagenomic Species Pan-genomes

Core, accessory, shared core and shared accessory genes associated with a core seed were assembled in a MSP.

Core genes were compared to the core seed representative and sorted by decreasing $p_{nr}$ to highlight those the most suitable for taxonomic profiling. In each class except core, a clustering procedure similar to the one used to create seeds was run to identify modules of co-occurring genes that may be interpreted as functional units, i.e. operons. Unclustered genes were saved as singleton modules.

### 2.4 Implementation

MSPminer is implemented in C++ and uses the OpenMP framework to take advantage of multi-core processors. Particular attention was paid to generate reproducible results. Large datasets with millions of genes and thousands of samples can be processed in just a few hours on a single node server.

### 2.5 Simulated dataset

For evaluation purposes, we generated abundance tables simulating the counts of genes from a single virtual species. The pan-genome of this species consisted in 1000 core genes detected in all strains and 6000 accessory genes present only in some of them. Gene lengths were randomly drawn between 100 bp and 5000 bp. The prevalence of accessory genes was randomly drawn between 2.5% and 99.5%.

In a first simulation used to evaluate MSPminer ability to recover a species pan-genome, 200 samples containing each a single strain of the species were generated. The sequencing coverage of a strain in a sample was drawn from a uniform law (min = 0.6, max = 20) and read length was set to 100 bp. In a given sample, the theoretical number of reads mapped on a gene was calculated according to the gene length, the strain coverage and the presence or not of the gene in the strain. Finally, the observed gene counts were drawn from Poisson distributions with means equal to theoretical counts.

In the second simulation used to evaluate the robust measure, outliers were added by multiplying observed counts of each gene by either ¼, ⅓, 2, 3 or 4 in 5%, 10% and 20% of the samples were it was present.

Next, we progressively decreased the number of samples where the species was detected (200, 100 and 50) to apprehend the impact of this parameter on the completeness of MSPs.

Finally, we simulated samples carrying two strains of the species where the dominant strain is 5 to 10 times more abundant than the subdominant one as observed in fecal samples (Truong *et al.*, 2017).

## 3 Results

### 3.1 Evaluation on simulated data

#### 3.1.1 Evaluation of the measures of proportionality

**Fig. 4.**
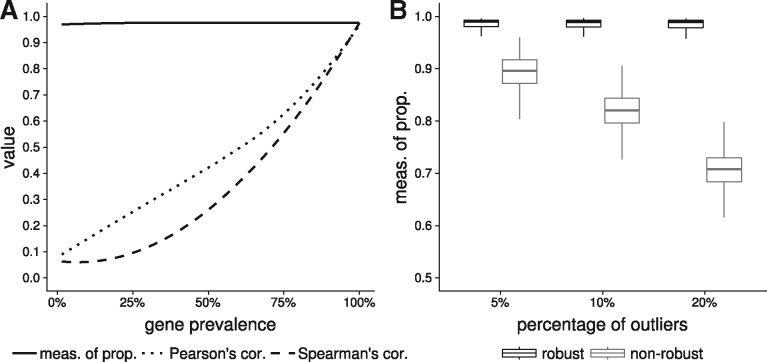
Evaluation of the measures of proportionality. (**A**) Comparison of the Pearson’s correlation coefficient, the Spearman’s correlation coefficient and the proposed measure of proportionality to detect an association between the median abundance vector of the core genes of the simulated species and the abundance vectors of each of its genes. The x-axis corresponds to the percentage of samples where a gene is detected and the y-axis corresponds to the intensity of the relationship between the compared vectors. The closer the value is to 1, the stronger the intensity of the relationship. (**B**) Comparison of the performances of the robust (black) and the non-robust (grey) measures of proportionality to detect a relationship between the noisy abundance vector of each gene of the simulated species and the outlier-free median abundance vector of its core genes. The proportion of outliers is gradually increased to 5%, 10% and 20%

First, we simulated the abundance table of a species across 200 samples to compare the performance of Pearson’s correlation coefficient, Spearman’s correlation coefficient and the proposed measure of proportionality ($p_{nr}$) for detecting a relation between the abundance profile of the species core genome and all its genes including accessories. Pearson’s and Spearman’s correlation coefficients decreased with the prevalence of the tested gene, while the proposed measure remained high, as it only uses samples where both the species core and the tested gene are detected (Fig. 4A). Therefore, the association between core genes and many accessory genes will be missed using the correlation coefficients. However, accessory genes observed in similar subsets of samples could be grouped into small distinct clusters as their abundance profiles should be correlated. Our simulations also show that $p_{nr}$ is more sensitive to species with highly variable coverage and on long genes as their counts are higher and less dispersed (Supplementary Fig. S5).

Then, we compared the robust measure of proportionality ($p_{r}$) against its non-robust counterpart ($p_{nr}$) by adding an increasing percentage of outliers to the genes abundance profiles. For a given percentage of outliers, each of these genes was compared to the outlier-free abundance profile of the core. This simulation showed that $p_{nr}$ decreases when the percentage of outliers increases whereas ($p_{r}$) remains high, demonstrating that proportionality is still detected despite the presence of samples with inconsistent counts (Fig. 4B).

#### 3.1.2 Evaluation of the clustering algorithm

**Fig. 5.**
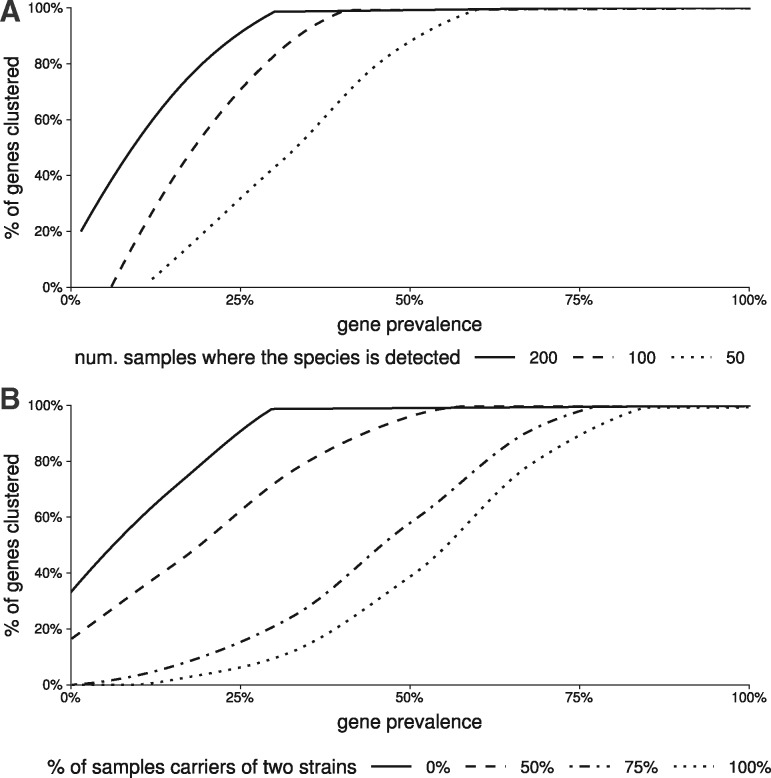
Evaluation of the clustering algorithm. (**A**) Impact of number of samples where the simulated species is detected on clustering. (**B**) Impact of strain mixture on clustering

Next, we tested if the number of samples where the species was detected had an influence on the completion of its corresponding MSP. Although this parameter did not impact the clustering of core and prevalent accessory genes, rarer accessory genes were grouped in the MSP only when the species was detected in a sufficiently large number of samples (Fig. 5A).

Finally, we explored the impact of mixture of multiple strains of the same species in samples. When occasional, strains mixture had little impact on clustering. If it was more frequent, many accessory genes of low or medium prevalence were missed (Fig. 5B). However, strains mixture might have less impact on the clustering performance. When it occurred, we considered that the presence of a gene in one strain was independent of its presence in the other. Yet, the low nucleotide divergence frequently observed between strains present in the same fecal sample suggests that they may have similar gene content (Truong *et al.*, 2017).

### 3.2 Application to the study of the human gut microbiota

We applied MSPminer to the largest publicly available gene abundance table provided with the Integrated Gene Catalog of the human gut microbiome (Li *et al.*, 2014). In this table, 9 879 896 genes are quantified across 1267 stool samples from individuals of various geographical origin (Europe, USA and China) and diverse health status (healthy, obese, diabetic, with inflammatory bowel disease etc.). 6 971 229 genes (70.6%) with counts greater than 6 in at least 3 samples were kept. Among these, 3 288 928 (47.2%) were organized into 1661 MSPs (Supplementary Table S1).

#### 3.2.1 Census of universal single copy marker genes

To check that MSPs correspond to real microbial species and evaluate the completeness of their core genomes, we identified 40 universal single copy marker genes (SCM) in the gene catalog (Sunagawa *et al.*, 2013). 84% of the SCMs detected in at least three samples were assigned to MSPs, indicating that MSPs capture a large proportion of the biological signal at species level. 915 MSPs (55%) had at least 30 SCM and 406 (24%) had all of them (Supplementary Table S2). As housekeeping genes, SCMs are essential to the microbe survival and expected among core genes. Indeed, 93% of the SCMs were core genes in their respective MSP and 70% of non-core SCMs were accessory genes of high prevalence (≥90%). This shows that the heuristic used for the classification of genes is reliable.

#### 3.2.2 Precision

We evaluated the precision of MSPminer by calculating in MSPs the fraction of genes assigned to the dominant species (Supplementary Table S4A). Apart from unassigned genes, the taxonomic consistency was very high for all gene categories (mean > 98%) except shared accessory genes (mean = 83.3%). Remarkably, some MSPs such as those representative of *Bacteroides plebeius*, *Ruminococcus bicirculans* and *Eubacterium eligens* had many unknown accessory genes (resp. 2888, 2821 and 2399) which is coherent with the low number of genomes available for these species. On average, 80% of these novel accessory genes were validated by performing the taxonomic annotation of the contigs they derived from. The remaining genes were found in unassigned contigs or contigs carrying only one gene. Conversely, 99% of the genes of the MSP representative of *Escherichia coli* (msp_0005) were annotated as thousands of references are available for this species.

#### 3.2.3 Sensitivity

Then, we aligned 3143 genomes representative of 322 species of the human gut microbiome against the IGC catalog. For each genome, we defined the sensitivity as the number of its genes grouped in the most representative MSP divided by the total number of its genes found in the catalog (Supplementary Table S4C). Overall, the sensitivity weighted by the number of genomes per species was high (median = 77%). Interestingly, genes grouped in MSPs were significantly longer than those that were not (median length of 975 bp versus 670 bp, Wilcoxon rank-sum test *P*-value = 0). More specifically, genomes of 1127 human gut-associated *E. coli* strains were well covered by the msp_0005 (mean = 83.4%). 95% of the core genes of genomes were also tagged as core in the MSP which shows again the robustness of the classification. However, 32 078 genes from the IGC catalog detected in *E. coli* genomes were missing in the msp_0005. 85% of these genes were present in less than 5% of the metagenomic samples where *E. coli* was detected, indicating that MSPminer misses rarest accessory genes which can be very numerous.

#### 3.2.4 Comparison to the canopy clustering algorithm

MSPminer was compared to the Canopy clustering algorithm (Nielsen *et al.*, 2014) which is the only gene binning tool publicly available. Both tools were applied to the metagenomic dataset described above using default parameters (Supplementary Methods). In total, MSPminer grouped 17.8% more genes than Canopy (3 288 928 versus 2 704 552) although MSPminer had a more stringent gene selection criterion (6 971 229 versus 7 304 439 genes processed). Both tools had a very high precision (mean > 98%) but MSPminer brought a significant gain in sensitivity (median: 77% versus 62%) (Supplementary Table S4). Remarkably, Canopy produced more objects with at least 150 genes than MSPminer (2010 CAGs versus 1661 MSPs) as it splits some species (e.g. *E. coli*) into multiple clusters. In contrast, MSPminer generated one MSP per species which improves downstream statistical analysis. Finally, MSPminer achieved better computing performance than Canopy (wall time: 2 h 40 min versus 42 h) while consuming less memory (peak memory: 74Go versus 231Go).

#### 3.2.5 MSPs taxonomy and phylogeny

642 MSPs (38.7%) could be annotated at species level, 315 (19.0%) at genus level, 525 (31.6%) at a higher taxonomic level from family to superkingdom and the remaining 179 (10.8%) could not be annotated (Supplementary Table S3C). Among the 642 MSPs with a species-level assignment, 303 corresponded to taxa validated by the *International Code of Nomenclature of Bacteria*, 56 matched genomes with imprecise taxonomy (e.g. sp., cf.) and 283 were metagenome-assembled genomes*.* In the end, most MSPs assigned to well-defined species matched RefSeq reference genomes. Combined with phylogenetic analysis, these results reveal that the majority of MSPs correspond to species that have not been isolated or sequenced so far (Supplementary Fig. S6).

Among the annotated MSPs, one corresponded to *Homo sapiens*, four were unicellular eukaryotes of the genus *Blastocystis*, eight were *Archaea* and the remaining 99% were *Bacteria* represented predominantly by the phyla *Firmicutes* (1016 MSPs), *Bacteroidetes* (263 MSPs), *Proteobacteria* (94 MSPs) and *Actinobacteria* (46 MSPs).

Interestingly, 15 species were represented by multiple MSPs such as *Faecalibacterium prausnitzii* (7 MSPs), *Bacteroides fragilis* (2 MSPs) or *Methanobrevibacter smithii* (2 MSPs) (Supplementary Table S3D). In these cases, one of the MSPs matched the species reference genome and the other MSPs matched other genomes only. The low Average Nucleotide Identity (ANI) between these genomes and the species reference suggests that they actually belong to distinct species.

Conversely, 8 MSPs were attributed to reference genomes of different species (Supplementary Table S3E). For all cases, the comparison of the reference genomes revealed an ANI > 96%, suggesting that they actually belonged to the same species despite distinct names were attributed.

Among the 3813 genomes that matched MSPs annotated at species level, 369 with imprecise taxonomy could be reassigned to well-defined species and 581 appeared misannotated or contaminated (Supplementary Table S3B).

#### 3.2.6 MSPs content

Most MSPs were small (median number of genes = 1821) even if 51 had more than 5000 genes (Supplementary Fig. S7 and Supplementary Table S2). As expected, a strong positive correlation (Pearson’s *r* = 0.78) between the total number of genes in a MSP and its number of accessory genes was observed. Interestingly, four outliers corresponding to the unicellular eukaryotes previously described had a high number of core genes and few accessory genes. This suggests that Eukaryotic genomes have a larger number of genes and a lower gene content variability than Prokaryotes. Among the MSPs with the more accessory genes, many corresponded to species reported as highly variable such as *Klebsiella pneumoniae* (Holt *et al.*, 2015) or *Clostridium bolteae* (Dehoux *et al.*, 2016). As previously observed in population genomics studies comparing multiple strains of the same species (Koonin and Wolf, 2008), the prevalence of accessory genes in MSPs often follows a bimodal distribution showing either a high or low prevalence but rarely intermediate (Supplementary Fig. S8).

#### 3.2.7 MSPs prevalence

Most MSPs were very rare as 596 (35.9%) were detected in less than 1% of samples and 1110 (66.2%) in less than 5%. Only 82 MSPs (4.9%) were detected in at least half of the samples showing that the common microbial core of the human gut microbiota is limited to a few dozen species (Supplementary Table S2). MSPs annotated at species level were significantly more frequent than those with less precise annotation (median prevalence: 5.4% versus 1.7%, *P*-value = 1.4.10^−21^ Wilcoxon rank-sum test) indicating that non-sequenced species are generally rarer. No clear relation between the prevalence of the MSPs and their mean abundance was found. However, two MSPs corresponding to *Bacteroides vulgatus* and *Bacteroides uniformis* were both very prevalent (detected in 97.5% and 94.0% of the samples, respectively) and very abundant (mean relative abundance of 7.3% and 4.1%, respectively). Interestingly, many rare MSPs assigned to the *Prevotella* genus were abundant in the few samples which carried them.

#### 3.2.8 MSPs quantification for biomarkers discovery

To demonstrate that MSPminer was useful for biomarkers discovery, we first looked for differentially abundant MSPs according to the geographical origin of samples (Supplementary Methods). We discovered 343 MSPs differentially abundant between Westerners and Chinese including 259 more abundant in Westerners and 84 in Chinese (Supplementary Table S5A). Among the discriminant MSPs, all those assigned to the *Proteobacteria* phylum (*Klebsiella pneumoniae*, *Escherichia coli* and *Bilophila wadsworthia*) were more abundant in Chinese which is consistent with previously published results (Li *et al.*, 2014). Interestingly, three MSPs assigned to *Faecalibacterium prausnitzii* were significant but two were more abundant in Westerners and the other in Chinese. In addition, we discovered 134 MSPs differentially abundant between Europeans and Americans of which 119 were more abundant among Europeans (Supplementary Table S5B). This result is consistent with previous studies showing lower gut microbiota diversity among Americans compared to Europeans (Sunagawa *et al.*, 2013).

Secondly, we used MSPs for strain-level analysis. To this end, we looked for accessory genes more frequent in samples of a given geographical origin (Supplementary Methods). We found 51 MSPs with at least 200 such accessory genes (Supplementary Table S5C). Some MSPs contained genes associated with sample origin while the abundance of their core was not, illustrating the complementarity of the two approaches.

## 4 Discussion

### 4.1 Identification of genes with proportional counts

MSPminer relies on a new robust measure ($p_{r}$) to detect genes with directly proportional counts. This relation more stringent than those assessed by Pearson’s or Spearman’s correlation coefficients was successfully used to reconstitute Metagenomic Species Pan-genomes of the human gut microbiota. In fact, most genes from sequenced genomes were grouped into a single MSP showing that direct proportionality is the most common relation between genes from the same biological entity.

However, MSPminer misses some genes for which counts are not ruled by this relation. Indeed, proportionality is disrupted when gene copy number varies across samples (Greenblum *et al.*, 2015), when a sample contains multiple strains of the same species (Truong *et al.*, 2017), when a gene is subject to horizontal gene transfer (Brito *et al.*, 2017), or when genes from closely related species are represented by the same reference after redundancy removal. Nevertheless, the first two cases have most likely a limited impact as the majority of strains tend to have the same gene copy numbers (Greenblum *et al.*, 2015) and samples often carry a dominant strain (Truong *et al.*, 2017). Regarding shared genes, their signals are a linear combination of the MSPs that carry them. Thus, they will be identified only if these MSPs are mostly detected in separate sets of samples.

### 4.2 Parameters impacting the quality of the MSPs

The quality of the MSPs is impacted by the upstream steps required for generating the gene counts table, as well as by the biological and ecological characteristics of the dataset. At the sequencing level, the number of reads (sequencing depth) generated for each sample impacts the detection and coverage of subdominant species, while read length affects the quality of the assembly and the ability to assign a read to a gene without ambiguity. At the bioinformatics level, assembly, gene prediction, gene redundancy removal, mapping and counting require expertise to select the most appropriate strategies, tools and parameters. Indeed, assemblers returning chimeric contigs which combine sequences from highly related species, inaccurate predictors generating truncated or merged genes, redundancy removal with a common threshold for all genes (95% of nucleotide identity) lead to genes of variable quality in catalogues. When quantifying genes, keeping only uniquely mapped reads underestimates the abundance of some genes whereas considering shared reads can generate false positives. As shown on simulated data and verified on a real metagenomic dataset, longer genes are more likely to be clustered in MSPs because they have greater and less dispersed counts. Finally, at the biology level, a high number of samples with varied phenotypes will improve the comprehensiveness and quality of MSPs. Indeed, as the number of samples grows, MSPminer will be able to identify rare species and assign rarer accessory genes to their respective MSPs. In addition, highly prevalent accessory genes will be reclassified from core to accessory as observed while sequencing an increasing number of strains of a species (Touchon *et al.*, 2009).

### 4.3 Applications

As illustrated in this paper, MSPs can be used for taxonomic profiling of human gut metagenomes. By using a dedicated pipeline (Kultima *et al.*, 2012), the sequencing reads need to be mapped on the IGC catalog to get the number times each gene was sequenced. Then, the aggregation of the core genes abundance profiles of each MSP allows accurate detection and quantification of microorganisms in samples up to species level. New MSPs will need to be built if those provided are not representative of the studied ecosystem.

Compared to methods relying on reference genomes (Truong *et al.*, 2015), information from unknown or non-sequenced species can be exploited. In addition, our method is not impacted by contaminated genomes or incorrect taxonomic annotation. Compared to methods quantifying a few dozen marker genes (Sunagawa *et al.*, 2013), MSPminer may improve the estimation of species abundance by automatically detecting among hundreds of core genes those with the greatest specificity and sensitivity.

Furthermore, in each MSP, one can build a presence/absence table of accessory genes to compare strains carried by individuals and discover biomarkers associated with specific functional traits such as pathogenicity. Finally, MSPminer provides microbial population genetics from large cohorts which can help culture-dependent methods prioritize species of greater interest, such as those with no reference genome available or with reference genomes distant from the strains present in metagenomic samples (Fodor *et al.*, 2012). When sequencing coverage allows, genomes of these species can be directly reconstituted from metagenomic assemblies by binning contigs carrying genes of the same MSP.

## Funding

This work was funded by Enterome, the ANRT (Association Nationale de la Recherche et de la Technologie) via the grant CIFRE 2014/0057 and INRA MetaGenoPolis via the grant ‘Investissements d'avenir’ ANR-11-DPBS-0001.

*Conflict of Interest*: none declared.

## Supplementary Material

Supplementary Data
