# Supplementary material for: MSPminer: abundance-based reconstitution of microbial pan-genomes from shotgun metagenomic data
Source: Bioinformatics. 2018 Sep 25;35(9):1544–52. doi: 10.1093/bioinformatics/bty830 (PMC6499236; doi:10.1093/bioinformatics/bty830)
Supplement: Supplementary Data [file bty830_supp.zip › bty830-suppl_data/bty830_Supplementary Information.pdf]

*Data and text mining*

# MSPminer: abundance-based reconstitution of microbial pan-genomes from shotgun metagenomic data

Florian Plaza Oñate[1,2*], Emmanuelle Le Chatelier[2], Mathieu Almeida[2], Alessandra C. L. Cervino[1], Franck Gauthier[2], Frédéric Magoulès[3], S. Dusko Ehrlich[2,4] and Matthieu Pichaud[1]

[1]Enterome, 94-96 Avenue Ledru Rollin, 75011 Paris, France, [2]MGP MetaGénoPolis, INRA, Université Paris-Saclay, 78350 Jouy en Josas, France, [3] Centrale Supélec, Université Paris Saclay, 9 rue Joliot Curie, 91190 Gif-sur-Yvette, France, [4]Centre for Host Microbiome Interactions, Dental Institute, King's College London, UK

*To whom correspondence should be addressed.

## 1 Supplementary Tables

**Supplementary Information**: Supplementary Methods and Supplementary Figures
**Supplementary Table 1**: TSV file listing the genes grouped in the 1 661 MSPs
**Supplementary Table 2**: XLS file describing the 1 661 MSPs (number of genes, number of universal marker genes, prevalence and abundance)
**Supplementary Table 3**: XLS file giving the taxonomic annotation of the 1 661 MSPs.
**Supplementary Table 4**: XLS file giving the sensibility and the specificity of Canopy and MSPminer.
**Supplementary Table 5**: XLS file listing the MSPs and the MSPs accessory genes associated with the geographic origin of samples.

## 2 Supplementary Methods

### 2.1 Taxonomic annotation

#### Annotation of the MSPs

MSPs taxonomic annotation was performed by aligning all core and accessory genes against nt and WGS (version of September 2017 restricted to the taxa Bacteria, Archaea, Fungi, Viruses and Blastocystis) using blastn (Altschul *et al.*, 1990) (version 2.7.1, task = megablast, word_size = 16). The 20 best hits for each gene were kept.

A species-level assignment was given if > 50% of the genes matched the RefSeq reference genome of a given species, with a mean identity ≥ 95% and mean gene length coverage ≥ 90%. The remaining MSPs were assigned to a higher taxonomic level (genus to superkingdom), if more than 50% of their genes had the same annotation. When necessary, the Average Nucleotide Identity between a pair of genomes was computed using OrthoANI (Lee *et al.*, 2016).

#### Reannotation of sequenced genomes

For MSPs annotated at the species level, other genome hits were considered. If the MSP core genes were found to be close to another genome with ≥ 90% of gene length coverage and ≥ 97.5% of identity, this genome was flagged as putatively originating from the same species.

### 2.2 Construction of the phylogenetic tree

40 universal phylogenetic markers genes were extracted from the 1 661 MSPs and 460 reference genomes with fetchMG (Sunagawa *et al.*, 2013). MSPs with less than 5 markers were discarded. Then, the markers were separately aligned with MUSCLE (Edgar, 2004). The 40 alignments were merged and trimmed with trimAl (Capella-Gutiérrez *et al.*, 2009). Finally, the phylogenetic tree was computed with FastTreeMP (Price *et al.*, 2010) and visualized with iTOL (Letunic and Bork, 2016).

### 2.3 Selection of genomes used for sensitivity evaluation

Genes from genomes deposited in the GenBank database (Nov. 2017) were aligned against the IGC catalog using blastn (identity ≥ 95% and coverage ≥ 90%). Genomes with at least 50% of their constituent genes detected in catalog were kept for further analysis.

### 2.4 Comparison to the Canopy clustering algorithm

The latest implementation of the Canopy clustering was downloaded at bitbucket.org/HeyHo/mgs-canopy-algorithm. MSPminer and Canopy were executed on a server with 2 E5-2690 CPUs (2x12 cores) running on the CentOS7 operating system.

### 2.5 Biomarkers discovery

#### Identification of MSPs associated with geographical origin

A two-tailed Wilcoxon rank-sum test was used on relative median abundance of the 30 best representative core genes of each MSP quantified across samples of the two populations tested. The obtained p-values were adjusted by the Benjamini-Hochberg procedure. In addition, the log2 ratio was computed between the median abundances of the MSP in the two populations tested. MSPs with an adjusted p-value inferior to $10^{-3}$ and a log2 ratio superior to 1 were considered significant

### Identification of accessory genes associated with geographical origin

Let $g_1$ be the raw median abundance of the 30 best representative core genes of a MSP and $g_2$ the raw abundance profile of one of its accessory genes to be tested. $g_1$ and $g_2$ were compared using the robust measure to estimate their coefficient of proportionality.

Then, we generated a 2x3 contingency table counting for each geographical origin (China, Europe, USA) the number of samples where the accessory gene was present or absent. For a sample *s*, the accessory gene was considered present if it was detected with the MSP core $\left(c_{1,s} \geq t_1 \wedge c_{2,s} \geq t_2\right)$ or absent if its count could be classified as a structural zero $\left(c_{1,s} \geq t_1 \wedge c_{2,s} = t_2\right)$. Samples that did not meet any of these two criteria were not considered.

Finally, a chi-squared test was performed on the contingency table. The accessory gene was considered as associated with geographical origin if the obtained p-value was inferior to $10^{-10}$.

## 3 Supplementary Figures

See following pages.

## References

Altschul,S.F. *et al.* (1990) Basic local alignment search tool. *J. Mol. Biol.*, **215**, 403–10.

Capella-Gutiérrez,S. *et al.* (2009) trimAl: A tool for automated alignment trimming in large-scale phylogenetic analyses. *Bioinformatics*, **25**, 1972–1973.

Edgar,R.C. (2004) MUSCLE: Multiple sequence alignment with high accuracy and high throughput. *Nucleic Acids Res.*, **32**, 1792–1797.

Lee,I. *et al.* (2016) OrthoANI: An improved algorithm and software for calculating average nucleotide identity. *Int. J. Syst. Evol. Microbiol.*, **66**, 1100–1103.

Letunic,I. and Bork,P. (2016) Interactive tree of life (iTOL) v3: an online tool for the display and annotation of phylogenetic and other trees. *Nucleic Acids Res.*, **44**, W242–W245.

Price,M.N. *et al.* (2010) FastTree 2 - Approximately maximum-likelihood trees for large alignments. *PLoS One*, **5**.

Sunagawa,S. *et al.* (2013) Metagenomic species profiling using universal phylogenetic marker genes. *Nat. Methods*, **10**, 1196–1199.

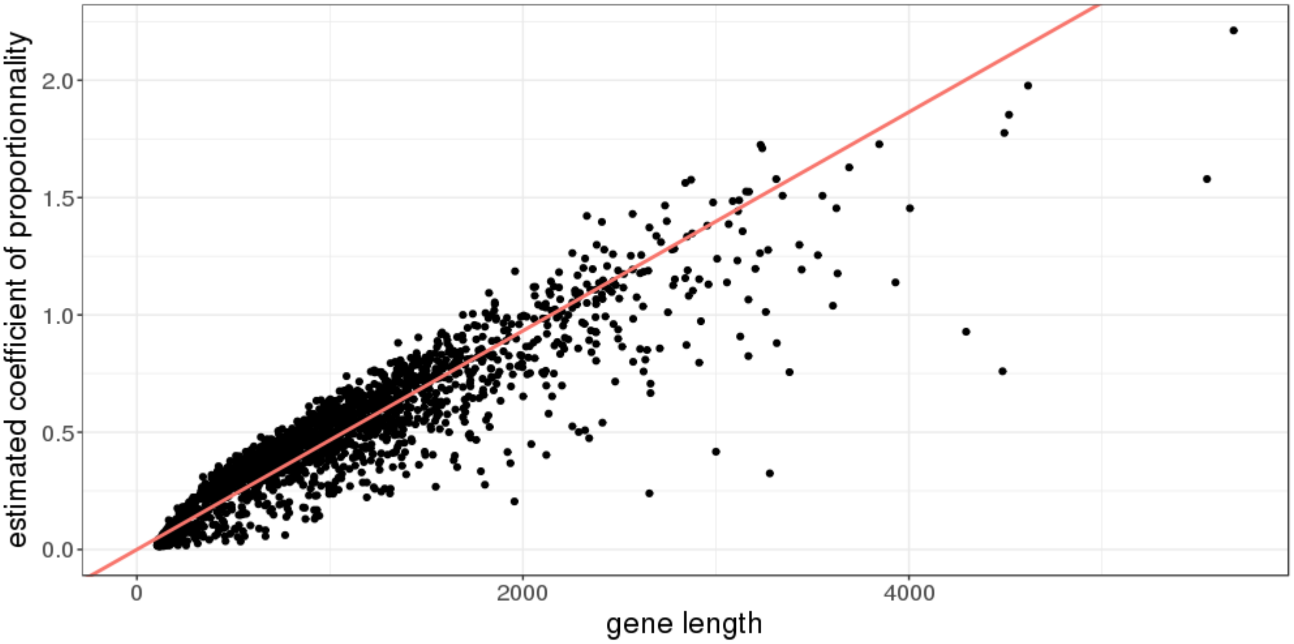


**Supplementary Fig. 1** Comparison of the length of the 1921 core genes of Parabacteroides distasonis (x-axis) and their respective coefficient of proportionality α (y-axis) .

Gene length is given in base pairs. The coefficient of proportionality α is estimated by comparing the abundance profile of each gene with the median abundance profile of the 30 best core genes in the 1267 samples of the IGC catalog. The red line corresponds to the trend computed with a robust linear regression with a null intercept.

The coefficient of proportionality is proportional to gene length (Pearson's r = 0.91, p-value = 0) However, points below the red line indicate that the sequencing coverage of some genes is lower than expected.

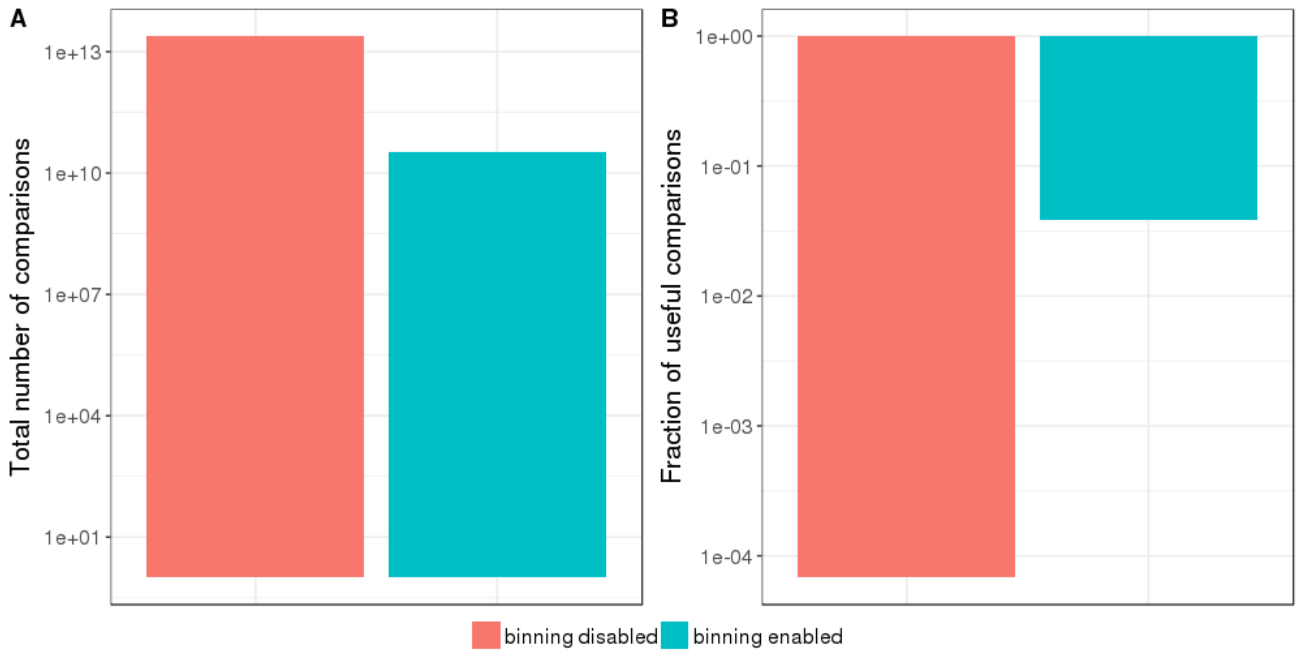


**Supplementary Fig. 2**: Performance of gene binning evaluated by the number of comparisons performed to group genes into seeds. 6 971 229 genes of the IGC catalog detected in at least 3 samples were considered.

A. Total number of comparisons performed by comparing all pairs of genes with or without binning. Here, genes binning divided the number of comparisons performed by 763.

B. Fraction of comparisons leading to the identification of clusters of co-abundant and co-occurring genes (seeds) with or without binning. The fraction without binning was estimated by drawing randomly one billion pairs of genes. Genes binning increased the fraction of useful comparisons by a factor 562.

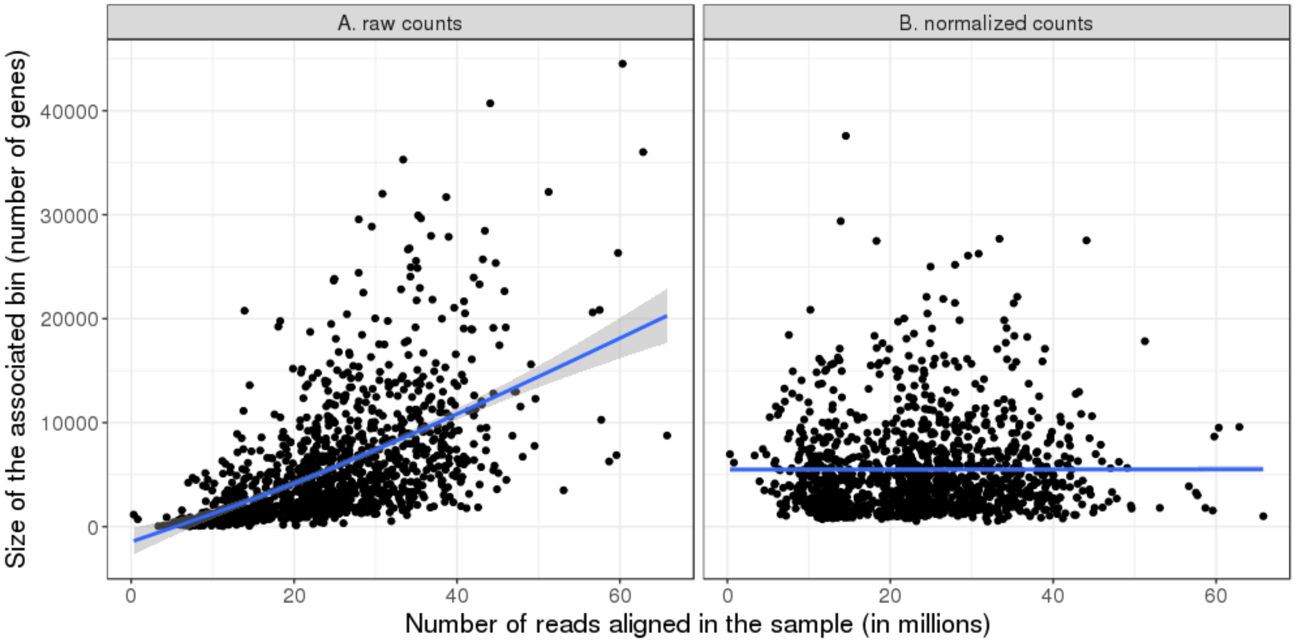


**Supplementary Fig. 3**: Impact of normalization on the size of the bins.

The x-axis corresponds the number of reads from a sample mapped on genes of the IGC catalog. The y-axis corresponds to the number of genes grouped in the bin of that sample.

A. Bins generated from raw counts. The blue trend line indicates that a bins contain more genes as the number of reads in the sample it represents is high. (Pearson's r =0.68, p-value < 2.1016)

B. Bins generated from counts normalized by the number of reads in samples. The blue trend line shows that there is no relation between the number of genes in a bin and the number of reads in the sample it represents. (Pearson's r =0.04, p-value=0.145)

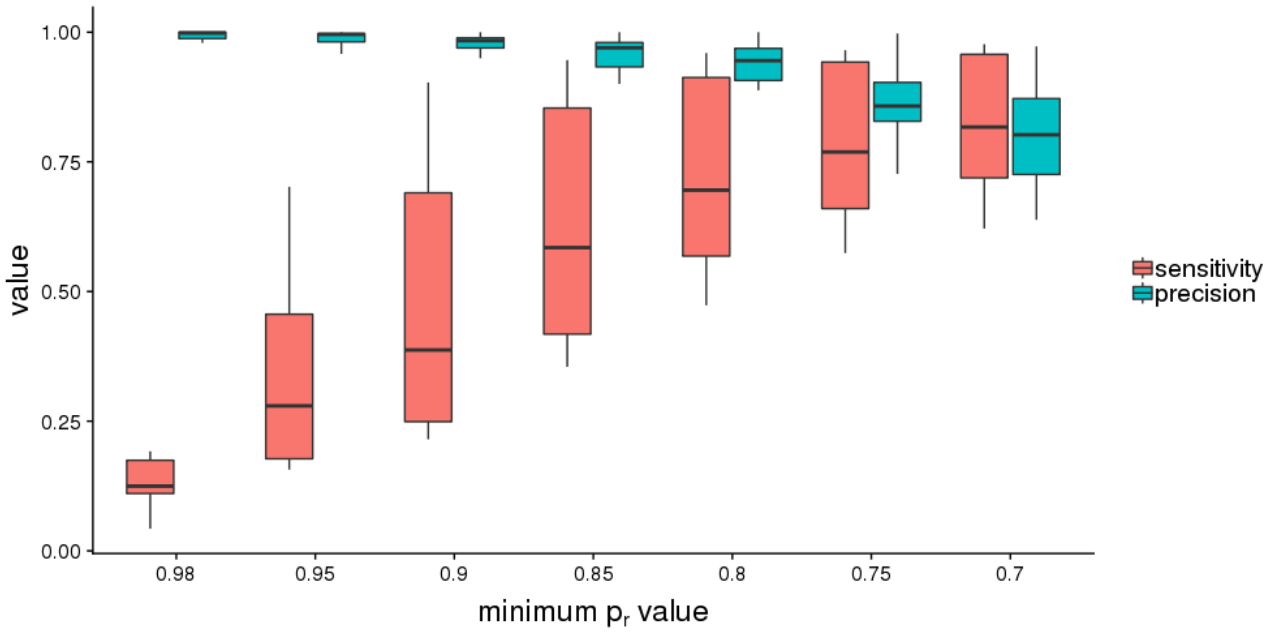


**Supplementary Fig. 4:** Quality of 8 MSPs by varying the minimum $p_r$ value for associating a gene with a core seed

With MSPminer, we generated the MSPs representative of 8 microbial species (c.f. table below) by varying the minimum $p_r$ value for associating genes from the IGC catalog with a core seed. The sensitivity is the fraction of genes from a species that are grouped in its corresponding MSP. The precision is the fraction of genes grouped in a MSP that are assigned to the expected species. We expect that most false detections are not previously unknown genes of these species as they have many sequenced genomes available.

We chose a minimal $p_r$ value equal to 0.8 because we observed a significant drop of precision by using lower thresholds.

| species | corresponding MSP | # sequenced genomes | prevalence in the IGC cohort |
|---|---|---|---|
| *Bacteroides thetaiotaomicron* | msp_0010 | 18 | 78.5% |
| *Parabacteroides distasonis* | msp_0011 | 16 | 89.3% |
| *Akkermansia muciniphila* | msp_0023 | 44 | 32.8% |
| *Ruminococcus gnavus* | msp_0061 | 18 | 39.2% |
| *Morganella morganii* | msp_0105 | 42 | 1.1% |
| *Bifidobacterium adolescentis* | msp_0255 | 26 | 46.9% |
| *Methanobrevibacter smithii* 1 | msp_0560 | 11 | 21.2% |
| *Lactobacillus fermentum* | msp_0950 | 45 | 1.3% |

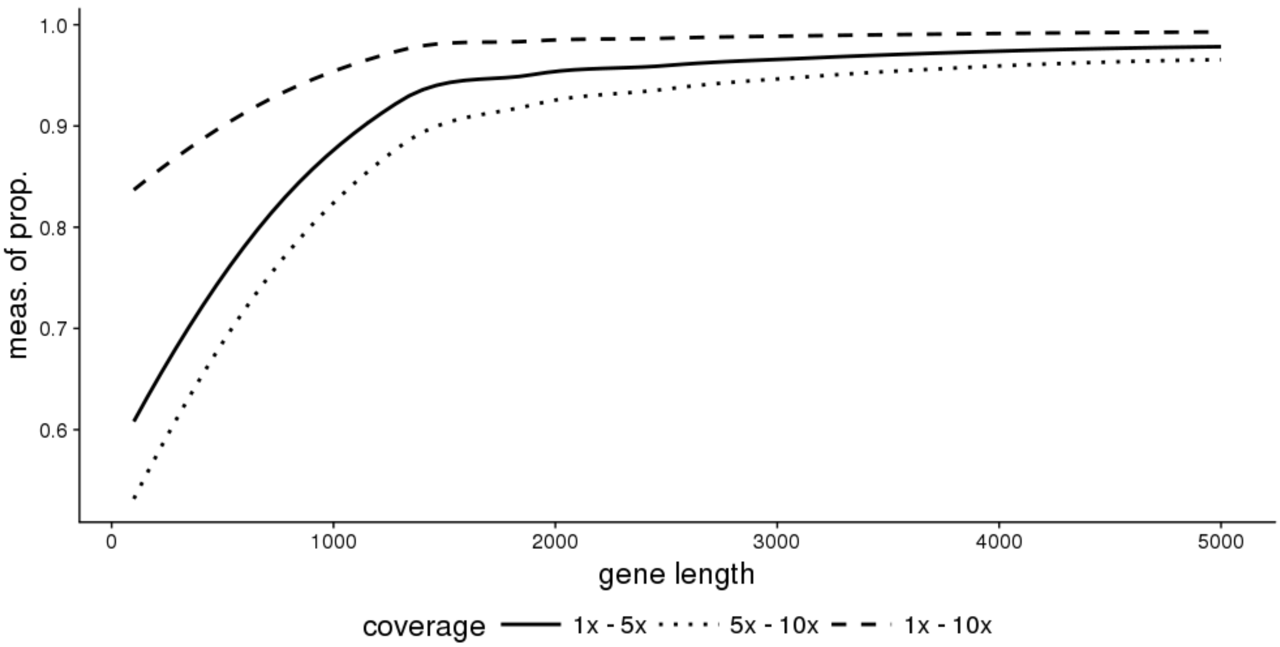


**Supplementary Fig. 5**: Impact of gene length and sequencing coverage on the non-robust measure of proportionality

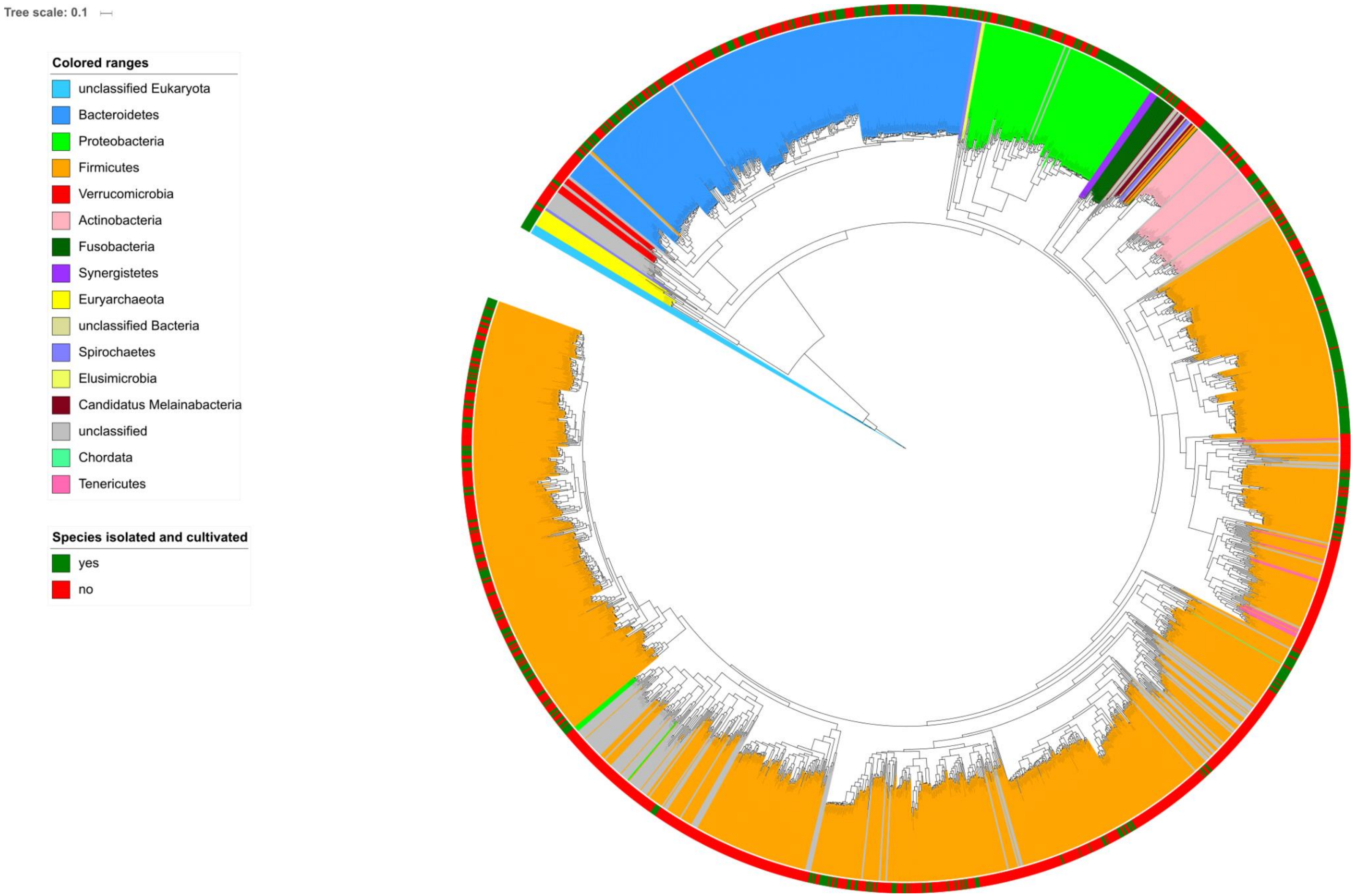


**Supplementary Fig. 6**: Phylogeny of the 1 661 MSPs and 460 genomes.

The inner colors correspond to the annotation of MSPs and genomes at phylum level. The outer colors indicate if the MSP is annotated at species level or not. White strips correspond to sequenced genomes. A high-resolution tree is available at https://itol.embl.de/tree/909224797355761524411234

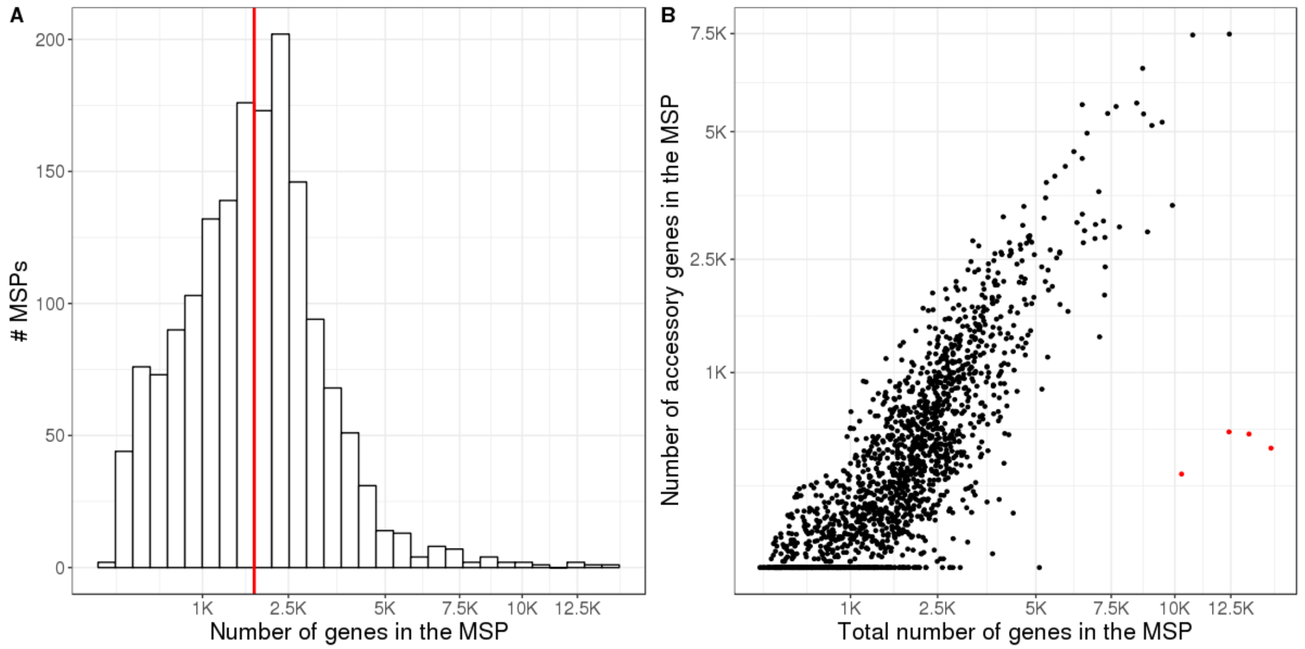


**Supplementary Fig. 7**: Size and gene content of the MSPs

A. Histogram representing the number of genes in the MSPs. The vertical red line corresponds to the median number of genes in the MSPs. (1 821 genes)

B. Number of accessory genes (y-axis) compared to the total number of genes in MSPs (x-axis). A strong correlation is observed between these two variables (Pearson's r = 0.78, p-value= 0). The four MSPs highlighted in red correspond to different species of the Blastocystis genus.

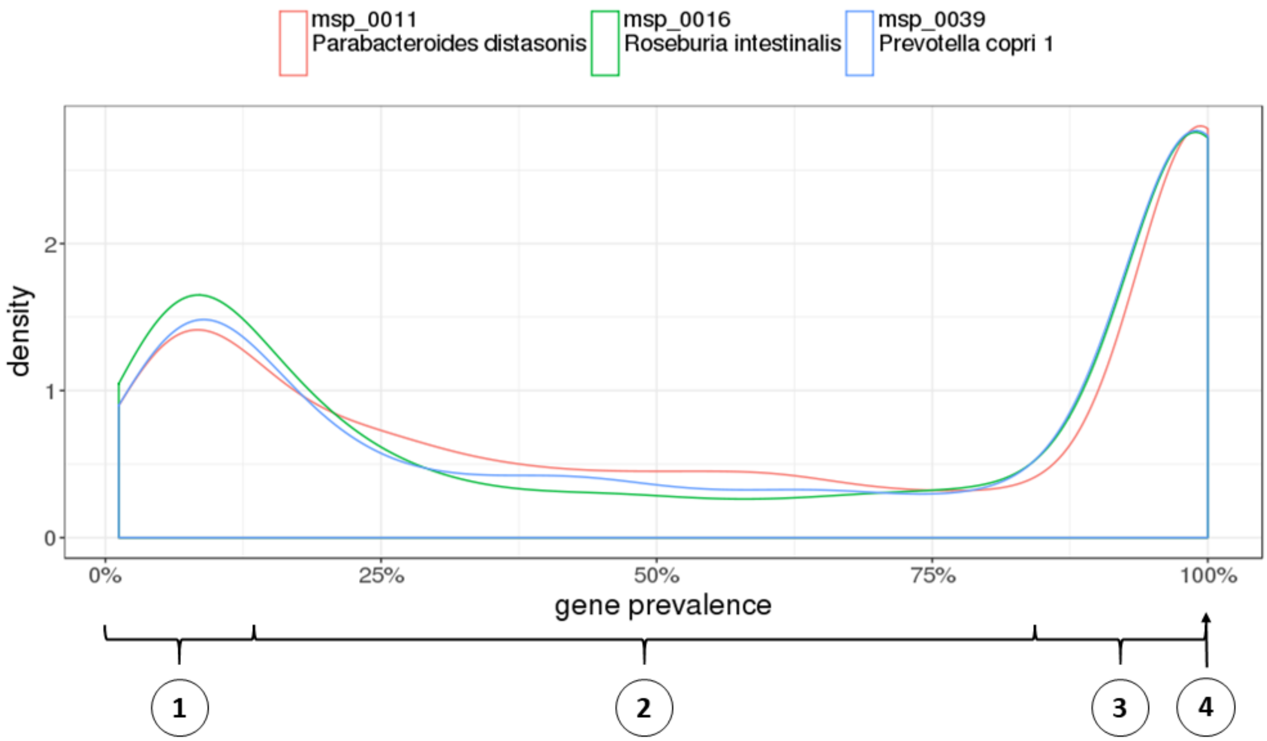


**Supplementary Fig. 8**: Prevalence of the genes of 3 MSPs among samples of the IGC catalog where their respective core are detected.

The x-axis corresponds to the prevalence of the gene and the y-axis to the proportion of genes with this prevalence.

This plot highlights 4 genes classes:

1. Cloud genes: rare accessory gene. Here, the proportion of cloud genes may be underestimated as rarest genes do not satisfy the co-occurrence criterion.
2. Shell genes: intermediate prevalence genes.
3. Soft core genes: highly prevalent accessory genes
4. (Hard) core genes: genes detected in all the samples where the species is present.
